# Growth Responses of Red-Leaf Lettuce to Temporal Spectral Changes

**DOI:** 10.3389/fpls.2020.571788

**Published:** 2020-10-22

**Authors:** Qingwu Meng, Erik S. Runkle

**Affiliations:** Controlled-Environment Lighting Laboratory, Department of Horticulture, Michigan State University, East Lansing, MI, United States

**Keywords:** controlled environment, dynamic lighting, LEDs, light quality, morphology, plant growth

## Abstract

Lighting is typically static for indoor production of leafy greens. However, temporal spectrum differentiation for distinct growth phases can potentially control age-specific desirable traits. Spectral effects can be persistent yet dynamic as plants mature, necessitating characterization of time-dependent responses. We grew red-leaf lettuce (*Lactuca sativa* L.) “Rouxai” in a growth room at 23°C and under a 20-h photoperiod created by warm-white (WW), blue (B; peak = 449 nm), green (G; peak = 526 nm), red (R; peak = 664 nm), and/or far-red (FR; peak = 733 nm) light-emitting diodes. From day 0 to 11, plants received six static lighting treatments with the same total photon flux density (400–800 nm): WW_180_, R_180_, B_20_R_160_, B_20_G_60_R_100_, B_20_R_100_FR_60_, or B_180_ (subscripts denote photon flux densities in μmol⋅m^–2^⋅s^–1^). On day 11, plants grown under each of the six treatments were transferred to all treatments, which created 36 temporal spectrum alternations. Plant growth, morphology, and coloration were measured on days 11 and 25. Increasing B radiation from 0 to 100% in static treatments decreased shoot fresh and dry weights and increased foliage redness of seedlings and mature plants. Compared to B_20_R_160_, B_20_R_100_FR_60_ increased shoot fresh weight, but not dry weight, on both days. However, other phenotypic responses under static treatments changed over time. For example, leaf length under B_180_ was 35% lower on day 11 but similar on day 25 compared to that under R_180_. In the B_20_ background, substituting G_60_ for R radiation did not influence shoot weight on day 11 but decreased it by 19% on day 25. When plants were switched from one treatment to another on day 11, the treatments applied before day 11 influenced final shoot weight and, to a lesser extent, leaf length and foliage coloration on day 25. In comparison, effects of the treatments applied after day 11 were more pronounced. We conclude some phenotypic responses to light quality depend on time and sequential light quality treatments had cumulative effects on lettuce growth. The temporal complexity of spectral responses is critical in photobiological research and creates opportunities for time-specific spectrum delivery to optimize crop characteristics.

## Introduction

The spectral composition of lighting in controlled environments can regulate a wide range of commercially relevant crop traits such as harvestable yield, morphology, coloration, and nutritional quality (Carvalho and Folta, 2014a). Red (R; 600–700 nm) radiation is typically more effective at stimulating extension growth and biomass accumulation of leafy greens than blue (B; 400–500 nm) or B + R radiation (Ohashi-Kaneko et al., 2007; Son and Oh, 2013; Lee et al., 2014). In contrast, B radiation generally suppresses extension growth (Cope et al., 2014; Wollaeger and Runkle, 2014) but stimulates production of bioactive compounds (Son and Oh, 2013; Lee et al., 2014; Kopsell et al., 2015). Green (G; 500–600 nm) radiation penetrates deep in the leaf and crop canopy to promote photosynthesis (Terashima et al., 2009; Brodersen and Vogelmann, 2010). Far-red (FR; 700–800 nm) radiation can induce shade-avoidance symptoms (Cerdán and Chory, 2003; Meng and Runkle, 2019) and regulate anthocyanin production (Carvalho and Folta, 2014b). The combined effects of these wavebands on plant growth and development are often complicated by synergistic or antagonistic interactions. Characterization of these spectral effects on various edible crops has been advanced by research with adjustable arrays of multicolored light-emitting diodes (LEDs) in controlled environments.

Electric lighting is substituted for sunlight to provide photosynthetically active photons for indoor-grown leafy greens. It is generally static throughout the production cycle, whereas field-grown plants undergo fluctuations in light quality, intensity, and duration throughout the day and production cycle. Static lighting feeds constant energy to light-harvesting antennae of photosystem II and maintains steady electron transport and proton generation to produce NADPH and ATP, respectively, which are used in carbon fixation (Armbruster et al., 2014). In contrast, the dynamic nature of sunlight necessitates responsive and efficient photosynthetic acclimation through regulation of energy channeling and dissipation to maintain high photosynthetic efficiency (Demmig-Adams et al., 2012). In arabidopsis [*Arabidopsis thaliana* (L.) Heynh.], K^+^ efflux antiporter 3 mediated H^+^/K^+^ antiport to facilitate rapid restoration of photosystem II quantum efficiency after plants were transferred from high to low light or from darkness to low light (Armbruster et al., 2014). Such mechanisms allow plants to thrive in continuously changing light environments.

Switching from static to dynamic lighting for indoor crop production adds the temporal factor in crop responses to improve crop traits. Temporal spectrum differentiation can occur in large or small segments of the crop life cycle to elicit age-dependent, desirable attributes. For example, anthocyanin accumulation in red-leaf lettuce (*Lactuca sativa* L.) is unnecessary for seedlings but desirable for mature plants at harvest. It can be induced rapidly by ≥4 days of end-of-production supplemental lighting from B and/or R LEDs (Owen and Lopez, 2015; Gómez and Jiménez, 2020). In addition, R radiation induced excessive extension growth of lettuce “Crispa” seedlings but increased dry weight of mature plants compared to B or B + R radiation (Chen et al., 2014). Therefore, it could be potentially beneficial to produce compact seedlings under B or B + R radiation and then switch to R radiation to promote growth of mature plants. After the seedling phase, weekly progressive spectrum alternations of B and/or R radiation influenced shoot growth, morphology, and phytochemical accumulation of lettuce “Sunmang” (Son et al., 2017). A greater dose of B radiation increased secondary metabolite concentrations, whereas a greater dose of R radiation increased shoot weight and projected leaf area (Son et al., 2017). Changing the spectrum in shorter periods of plant development can also modulate final crop phenotypes. For example, 4-day sequential B, R, and/or FR lighting treatments influenced stem elongation, anthocyanin concentration, and antioxidant capacity of kale (*Brassica napus* L. var. *sabellica*) seedlings, showing strong plant plasticity in response to spectral changes (Carvalho and Folta, 2014b). Furthermore, staggering B and R radiation within the day increased shoot weight of romaine lettuce (*Lactuca sativa* L. var. *longifolia*) compared to simultaneous B + R radiation (Jishi et al., 2016).

Under changing light conditions, a light response can be transient or persistent. Examples of a transient light response include stomatal opening and phototropism under B radiation as well as increasing net photosynthesis with incremental increases in photon flux densities. These rapid responses are reversible after the light condition changes. On the other hand, a spectrum applied in an early developmental phase can have persistent and irreversible influence on subsequent phenotypic responses. For example, the addition of FR radiation to B + R radiation during seedling development of snapdragon (*Antirrhinum majus* L.) promoted flowering when plants were finished in a greenhouse environment (Park and Runkle, 2017). In addition, B or R radiation applied for 7 days after emergence influenced leaf area and shoot dry weight of lettuce “Grand Rapids” 16 or 42 days after emergence, irrespective of a switch to the opposite waveband on day 7 (Eskins et al., 1995). However, such sustained spectral effects were not observed in other lettuce studies with a fixed spectrum early in seeding development and varying spectra afterward (Johkan et al., 2010; Son and Oh, 2013). Furthermore, the influence of a spectrum on lettuce growth and morphology can vary with each developmental phase. For example, when applied day 10–17 after seed sow, B radiation decreased leaf area and shoot fresh weight of lettuce “Banchu Red Fire” on day 17 but increased them on day 45 compared to R radiation (Johkan et al., 2010). The discrepancies in these studies likely result from different genetic backgrounds, light intensities, and spectral contexts.

Here, we expanded static spectral combinations to include G, FR, and warm-white (WW) radiation and created a wide array of lighting treatments shifted temporally between the seedling and mature phases of indoor lettuce production. The objectives of this study were (1) to investigate how spectral treatments for lettuce seedlings influence phenotypes of mature plants grown under different spectra; (2) to compare lettuce growth under single wavebands, combinations of two or three wavebands, and WW radiation; and (3) to find temporal spectral combinations for desirable lettuce growth and morphology. We postulated that (1) the spectral effects during the seedling stage would persist through the mature phase, regardless of the finishing spectral environment; (2) substituting G radiation for R radiation would increase lettuce growth during the seedling stage but have little influence on growth of mature plants; and (3) B radiation alone would inhibit leaf expansion and dry weight during the seedling phase but promote them during the mature phase.

## Materials and Methods

### The Propagation Phase

This experiment was performed in a refrigerated walk-in growth room of the Controlled-Environment Lighting Laboratory (Michigan State University, East Lansing, MI). We chose lettuce to study because it is the most widely grown hydroponic crop in indoor vertical farms for its short stature, fast growth rate, and high value. Our previous studies showed generally similar growth responses of green- and red-leaf lettuce, so we studied red-leaf lettuce because of its unique foliage coloration in response to spectral alternations (Meng and Runkle, 2019; Meng et al., 2019). Seeds of red oakleaf lettuce “Rouxai” were obtained from a commercial seed producer (Johnny’s Selected Seeds, Winslow, ME, United States) and sown in a rockwool substrate with 200 2.5-cm-wide cubes per sheet (AO 25/40 Starter Plugs; Grodan, Milton, ON, Canada) on April 28 and 29, 2018 for two blocks. The substrate was presoaked in deionized water supplemented with diluted (1:31) 95–98% sulfuric acid (J.Y. Baker, Inc., Phillipsburg, NJ, United States), a water-soluble fertilizer (12N–4P_2_O_5_–16K_2_O RO Hydro FeED; JR Peters, Inc., Allentown, PA, United States), and magnesium sulfate (Epsom salt; Pennington Seed, Inc., Madison, GA, United States) to achieve a pH of 3.9 and an electrical conductivity of 1.6 mS⋅cm^–1^. The nutrient solution contained the following nutrients (in mg⋅L^–1^): 125 N, 42 P, 167 K, 73 Ca, 49 Mg, 39 S, 1.7 Fe, 0.52 Mn, 0.56 Zn, 0.13 B, 0.47 Cu, and 0.13 Mo. Seed trays were covered with transparent humidity domes and placed under six different lighting treatments, each at a total photon flux density (TPFD; 400–800 nm) of 180 μmol⋅m^–2^⋅s^–1^ with a 20-h photoperiod. Air temperature was set at 20°C from April 28 to 30, 2018 and increased to 23°C for the remainder of the experiment. From day 1 to 11, seedlings were subirrigated as needed using the same nutrient solution with a pH of 5.8 adjusted with potassium bicarbonate. The humidity domes were removed on May 3, 2018 for both blocks.

### The Production Phase

On day 11, when the second true leaf was expanding, seedlings in rockwool cubes were transplanted into 36-cell rafts (36 2.5-cm-wide holes on each lightweight raft measuring 60.9 cm × 121.9 cm × 2.5 cm; Beaver Plastics, Ltd., Acheson, AB, Canada) floating in flood tables (1.22 m × 0.61 m × 0.18 m; Active Aqua AAHR24W; Hydrofarm, Petaluma, CA, United States) on three-tier racks (Indoor Harvest, Houston, TX, United States). Plants were spaced 20 cm apart horizontally and 15 cm apart diagonally. The recirculating nutrient solution was mixed as described for seedlings to provide the following nutrients (in mg⋅L^–1^): 150 N, 50 P, 200 K, 88 Ca, 58 Mg, 47 S, 2.1 Fe, 0.63 Mn, 0.68 Zn, 0.15 B, 0.56 Cu, and 0.15 Mo. It was oxygenated with a circular air stone (20.3 × 2.5 cm; Active Aqua AS8RD; Hydrofarm) connected to a 60-W air pump (Active Aqua AAPA70L; Hydrofarm). The pH, electrical conductivity, and temperature of the nutrient solution for each lighting canopy were measured daily using a portable pH and electrical conductivity meter (HI9814; Hanna Instruments, Woonsocket, RI, United States) (Table 1). Potassium bicarbonate was used to increase pH when it dropped below 5.5.

**TABLE 1:** The pH, electrical conductivity, and water temperature (mean ± standard deviation) of nutrient solutions for six lighting treatment plots in two blocks during the lettuce production phase.

Lighting treatment	pH		Electrical conductivity (mS⋅cm^–1^)		Water temperature (°C)	
	Block 1	Block 2	Block 1	Block 2	Block 1	Block 2
WW_180_	6.0 ± 1.0	6.2 ± 0.9	1.8 ± 0.0	1.8 ± 0.1	23.7 ± 0.3	23.3 ± 0.3
R_180_	6.1 ± 1.0	6.3 ± 0.9	1.9 ± 0.1	1.7 ± 0.1	22.9 ± 0.2	23.9 ± 0.4
B_20_R_160_	6.1 ± 1.0	6.3 ± 0.9	1.9 ± 0.1	1.7 ± 0.1	22.9 ± 0.2	23.9 ± 0.4
B_20_G_60_R_100_	6.1 ± 1.0	6.3 ± 0.9	1.9 ± 0.1	1.7 ± 0.1	22.9 ± 0.2	23.9 ± 0.4
B_20_R_100_FR_60_	6.0 ± 1.0	6.2 ± 0.9	1.8 ± 0.0	1.8 ± 0.1	23.7 ± 0.3	23.3 ± 0.3
B_180_	6.0 ± 1.0	6.2 ± 0.9	1.8 ± 0.0	1.8 ± 0.1	23.7 ± 0.3	23.3 ± 0.3

*Plants were grown under warm-white (WW) or mixed blue (B; 400–500 nm), green (G; 500–600 nm), red (R; 600–700 nm), and far-red (FR; 700–800 nm) light-emitting diodes (LEDs). The number following each LED type is its respective photon flux density in μmol⋅m^–2^⋅s^–1^.*

### Environmental Conditions

Temperature in the growth room was regulated with an industrial ventilation and air-conditioning unit (HBH030A3C20CRS; Heat Controller, LLC., Jackson, MI, United States) connected to a wireless thermostat (Honeywell International, Inc., Morris Plains, NJ, United States). The deep-flow hydroponic system was equipped with two light quantum sensors (LI-190R; LI-COR, Inc., Lincoln, NE, United States), two thermocouples (0.13-mm type E; Omega Engineering, Inc., Stamford, CT, United States), two infrared sensors (OS36-01-K-80F; Omega Engineering, Inc.), a CO_2_ transmitter (GMD20; Vaisala, Inc., Louisville, CO, United States), and a relative humidity and temperature probe (HMP110; Vaisala, Inc.). All sensors were connected to a datalogger (CR1000; Campbell Scientific, Inc., Logan, UT, United States) with a multiplexer (AM16/32B; Campbell Scientific, Inc.), which recorded environmental parameters every 10 s and logged hourly averages using computer software (LoggerNet; Campbell Scientific, Inc.). The air temperature, canopy temperature, CO_2_ concentration, and relatively humidity throughout the experiment (mean ± standard deviation) were 22.5 ± 1.0°C, 24.1 ± 0.9°C, 392 ± 31 ppm, and 44 ± 8%, respectively.

### Lighting Treatments

**FIGURE 1:**
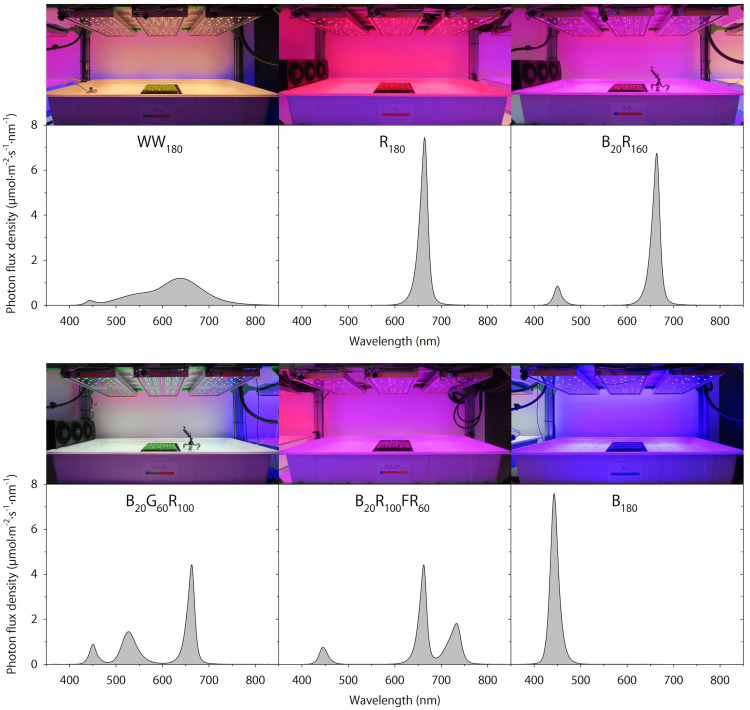
Spectral distributions of six lighting treatments delivered by warm-white (WW) or mixed blue (B; 400–500 nm), green (G; 500–600 nm), red (R; 600–700 nm), and far-red (FR; 700–800 nm) light-emitting diodes (LEDs). The number following each LED type is its respective photon flux density in μmol⋅m^–2^⋅s^–1^.

Seedlings were grown under WW_180_, R_180_, B_20_R_160_, B_20_G_60_R_100_, B_20_R_100_FR_60_, or B_180_ LEDs (PHYTOFY RL; OSRAM, Beverley, MA, United States), where the subscript following each LED type indicates its photon flux density (in μmol⋅m^–2^⋅s^–1^). The peak wavelengths of WW, B, G, R, and FR LEDs were 639, 449, 526, 664, and 733 nm, respectively. The outputs of seven color channels, including five used in this study, in each LED fixture were independently controlled with software (Spartan Control Software; OSRAM). The specifications, layout, and positioning of the LED fixtures were as described by Meng et al. (2019). Spectra were measured at seven locations at plant canopy of each lighting treatment using a portable spectroradiometer (PS200; Apogee Instruments, Inc., Logan, UT, United States) (Figure 1). The single-band photon flux densities, photosynthetic photon flux density (PPFD; 400–700 nm), TPFD, yield photon flux density [YPFD, an integrated value based on relative quantum efficiency (McCree, 1972) and spectral data], phytochrome photoequilibrium [PPE, an estimated value based on phytochrome absorption coefficients and spectra data (Sager et al., 1988)], ratio of B to R radiation (B:R), and ratio of R to FR radiation (R:FR) for each lighting treatment were subsequently calculated (Table 2). To study the temporal effects of light quality, lighting treatments were switched between the propagation phase (day 0–11) and the production phase (day 11–25). Seedlings grown under each of the six lighting treatments were transferred to all six lighting treatments on day 11. This created a total of 36 unique temporal lighting combinations, six of which were static (without transfers) throughout the experiment (Table 3).

**TABLE 2:** Spectral characteristics of six lighting treatments delivered by warm-white (WW) or mixed blue (B; 400–500 nm), green (G; 500–600 nm), red (R; 600–700 nm), and far-red (FR; 700–800 nm) light-emitting diodes (LEDs).

	LED lighting treatment					
	WW_180_	R_180_	B_20_R_160_	B_20_G_60_R_100_	B_20_R_100_FR_60_	B_180_
**Single-band photon flux density (μmol⋅m^–2^⋅s^–1^)**						
B	12.5	0.3	19.2	22.9	18.8	178.4
G	52.8	0.7	0.7	59.7	0.8	0.9
R	98.1	176.9	158.5	99.4	102.2	0.5
FR	18.1	2.1	1.9	1.3	60.7	0.1
**Integrated photon flux density (μmol⋅m^–2^⋅s^–1^)**						
PPFD	163.3	177.9	178.4	181.9	121.8	179.7
TPFD	181.4	180.0	180.3	183.2	182.4	179.9
YPFD	149.5	165.0	162.1	156.5	119.3	134.4
**Radiation ratio**						
B:R	0.13	0.00	0.12	0.23	0.18	386.80
R:FR	5.42	83.96	82.98	76.19	1.68	3.49
PPE	0.829	0.882	0.880	0.878	0.764	0.480

*The number following each LED type is its respective photon flux density in μmol⋅m^–2^⋅s^–1^. Integrated parameters include the photosynthetic photon flux density (PPFD; 400–700 nm), the total photon flux density (TPFD; 400–800 nm), and the yield photon flux density [YPFD; the product of relative quantum efficiency (McCree, 1972) and spectral data from 300 to 800 nm]. The estimated phytochrome photoequilibrium (PPE) was calculated as described by Sager et al. (1988).*

**TABLE 3:** Temporal lighting combinations during lettuce propagation and production.

Day 0–11 (propagation)	Day 11–25 (production)
WW_180_	WW_180_
	R_180_
	B_20_R_160_
	B_20_G_60_R_100_
	B_20_R_100_FR_60_
	B_180_
R_180_	WW_180_
	R_180_
	B_20_R_160_
	B_20_G_60_R_100_
	B_20_R_100_FR_60_
	B_180_
B_20_R_160_	WW_180_
	R_180_
	B_20_R_160_
	B_20_G_60_R_100_
	B_20_R_100_FR_60_
	B_180_
B_20_G_60_R_100_	WW_180_
	R_180_
	B_20_R_160_
	B_20_G_60_R_100_
	B_20_R_100_FR_60_
	B_180_
B_20_R_100_FR_60_	WW_180_
	R_180_
	B_20_R_160_
	B_20_G_60_R_100_
	B_20_R_100_FR_60_
	B_180_
B_180_	WW_180_
	R_180_
	B_20_R_160_
	B_20_G_60_R_100_
	B_20_R_100_FR_60_
	B_180_

*Plants were grown under static or alternate lighting treatments delivered by warm-white (WW) or mixed blue (B; 400–500 nm), green (G; 500–600 nm), red (R; 600–700 nm), and far-red (FR; 700–800 nm) light-emitting diodes (LEDs). The number following each LED type is its respective photon flux density in μmol⋅m^–2^⋅s^–1^.*

### Data Collection and Analysis

Shoot fresh and dry weights, leaf morphology, and coloration data were collected on ten young lettuce plants per block grown under each of the six static lighting treatments on day 11 and on eight mature lettuce plants per block grown under each of the 36 temporal lighting combinations on day 25. Shoot fresh weight was measured with an analytical balance (GR-200; A&D Store, Inc., Wood Dale, IL, United States) for young plants and a different one (GX-1000; A&D Store, Inc.) for mature plants based on capacities. Length of the fifth most mature true leaf was measured to quantify extension growth. The International Commission on Illumination Lab color space analysis was conducted on a representative leaf per plant using a colorimeter (Chroma Meter CR-400; Konica Minolta Sensing, Inc.). *L*^∗^, *a*^∗^, and *b*^∗^ indicate foliage brightness (ranging from 0 for black to 100 for diffuse white), greenness–redness (corresponding to negative–positive directions), and blueness–yellowness (corresponding to negative–positive directions), respectively. Subsequently, plants were dried in an oven (Blue M, Blue Island, IL) at 60°C for ≥5 days followed by dry weight measurements with the same analytical balances as for shoot fresh weight.

Data on young and mature lettuce plants were analyzed with the PROC MIXED procedure and Tukey’s honestly significant difference test (α = 0.05) in SAS (version 9.4; SAS Institute, Inc., Cary, NC, United States). Data from static treatments were analyzed as a randomized complete block design with two blocks (using opposite racks of the growth room), six static lighting treatments, and subsampling (*n* = 10), assuming fixed block effects. Data from alternate treatments were analyzed as a strip-split-plot design with two blocks, six whole-plot levels (post-transplant lighting treatments), six subplot levels (pre-transplant lighting treatments), and subsampling (*n* = 8), assuming fixed block effects. The split-plot design included whole plots arranged in a randomized complete block design.

## Results

### Static Lighting Treatments for Young and Mature Lettuce

**FIGURE 2:**
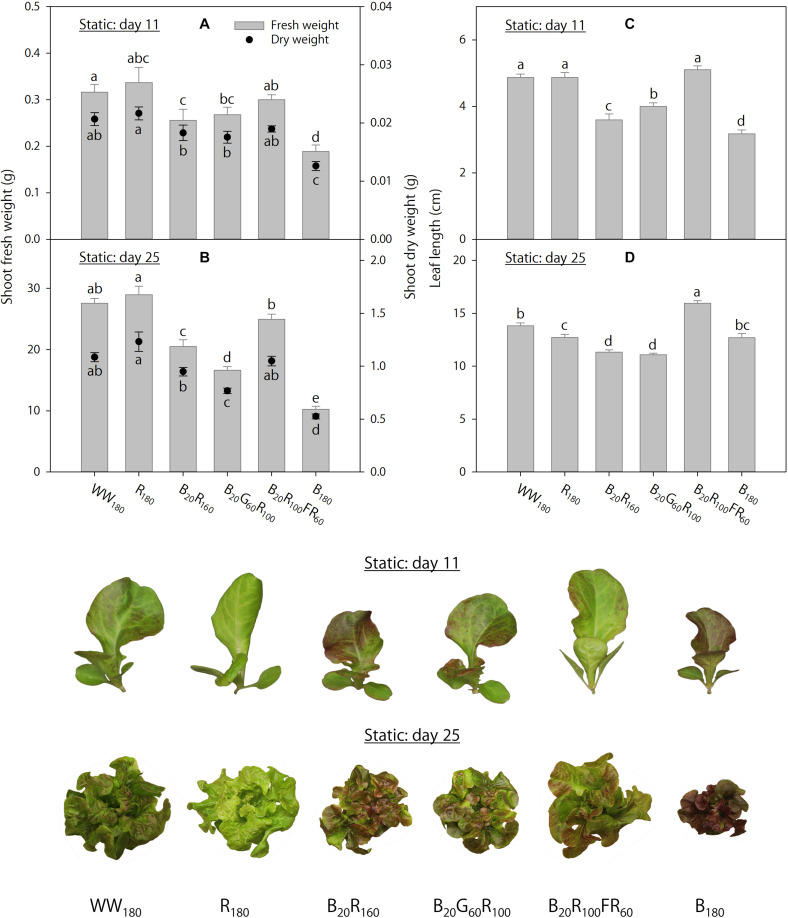
Shoot fresh and dry weights and leaf length on days 11 and 25 of lettuce “Rouxai” grown under six static lighting treatments delivered by warm-white (WW) or mixed blue (B; 400–500 nm), green (G; 500–600 nm), red (R; 600–700 nm), and far-red (FR; 700–800 nm) light-emitting diodes (LEDs). The number for each LED type is its photon flux density in μmol⋅m^–2^⋅s^–1^. Means followed by different letters within each parameter are significantly different based on Tukey’s honestly significant difference test (α = 0.05). Error bars show standard errors.

On day 11, shoot fresh weight was 40–44% lower, and shoot dry weight was 39–42% lower, under B_180_ than under WW_180_ and R_180_ (Figure 2A). Partial substitution of R radiation in B_20_R_160_ with 60 μmol⋅m^–2^⋅s^–1^ of G radiation (B_20_G_60_R_100_) or FR radiation (B_20_R_100_FR_60_) did not influence shoot dry weight, whereas the substitution with FR radiation increased shoot fresh weight by 18%. Substituting 20 μmol⋅m^–2^⋅s^–1^ of B radiation for R radiation (B_20_R_160_ versus R_180_) decreased shoot dry weight by 15%, but not shoot fresh weight. On day 25, increasing substitution of R radiation with B radiation decreased shoot fresh and dry weights (Figure 2B). Shoot fresh weight was 63–65% lower, and shoot dry weight was 52–57% lower, under B_180_ than under R_180_ or WW_180_. Substituting 60 μmol⋅m^–2^⋅s^–1^ of G radiation for R radiation in B_20_R_160_ decreased shoot fresh and dry weights by 19%. The same substitution with FR radiation increased shoot fresh weight by 22%, but not shoot dry weight.

On day 11, leaves were the longest under WW_180_, R_180_, and B_20_R_100_FR_60_ and the shortest under B_180_ (Figure 2C). Increasing substitution of R radiation with B radiation decreased leaf length. Substituting 60 μmol⋅m^–2^⋅s^–1^ of G or FR radiation for R radiation in B_20_R_160_ increased leaf length by 11 or 42%, respectively. On day 25, leaves were the longest under B_20_R_100_FR_60_ and the shortest under B_20_R_160_ and B_20_G_60_R_100_ (Figure 2D). Compared to WW_180_, leaves were 8% shorter under R_180_ and similar under B_180_. Although substituting 20 μmol⋅m^–2^⋅s^–1^ of B radiation for R radiation decreased leaf length by 11%, leaf length was similar under R_180_ and B_180_. Substituting 60 μmol⋅m^–2^⋅s^–1^ of FR radiation for R radiation in B_20_R_160_ increased leaf length by 41%, but the same substitution with G radiation did not influence it.

**FIGURE 3:**
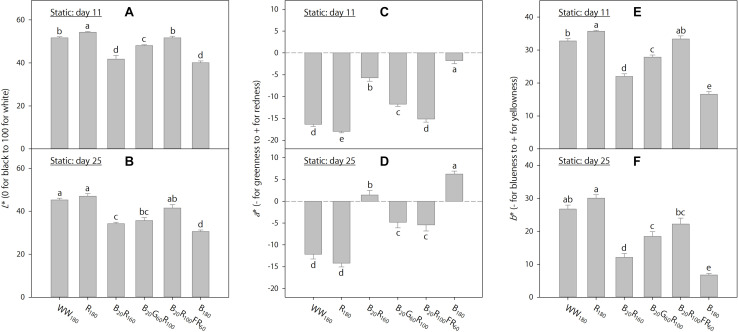
Lab color space parameters on days 11 and 25 of lettuce “Rouxai” grown under six static lighting treatments delivered by warm-white (WW) or mixed blue (B; 400–500 nm), green (G; 500–600 nm), red (R; 600–700 nm), and far-red (FR; 700–800 nm) light-emitting diodes (LEDs). The number for each LED type is its photon flux density in μmol⋅m^–2^⋅s^–1^. Means followed by different letters in each graph are significantly different based on Tukey’s honestly significant difference test (α = 0.05). Error bars show standard errors.

On day 11, foliage brightness (*L*^∗^) was the greatest under R_180_, followed by WW_180_ and B_20_R_100_FR_60_ (Figure 3A). Adding B radiation to R_180_ decreased brightness. Substituting R radiation in B_20_R_160_ with G or FR radiation increased brightness, especially with FR radiation. On day 25, leaves were the brightest under R_180_ and WW_180_ and the darkest under B_180_ (Figure 3B). Increasing substitution of R radiation with B radiation decreased brightness. Leaves were brighter when R radiation in B_20_R_160_ was substituted with FR radiation, but not G radiation.

On day 11, leaves were the least red (lowest *a*^∗^) and yellowest (highest *b*^∗^) under R_180_, followed by WW_180_ and B_20_R_100_FR_60_, and the reddest and least yellow under B_180_ (Figures 3C,E). The inclusion of B radiation in an R background increased redness and decreased yellowness, whereas the inclusion of G or FR radiation decreased redness and increased yellowness. At the same photon flux density, FR radiation reduced redness and increased yellowness more than G radiation. The *a*^∗^ and *b*^∗^ trends on day 25 were similar to those on day 11, except that there were no differences between R_180_ and WW_180_ or between B_20_G_60_R_100_ and B_20_R_100_FR_60_ on day 25 (Figures 3D,F).

### Temporal Lighting Combinations for Mature Lettuce

**FIGURE 4:**
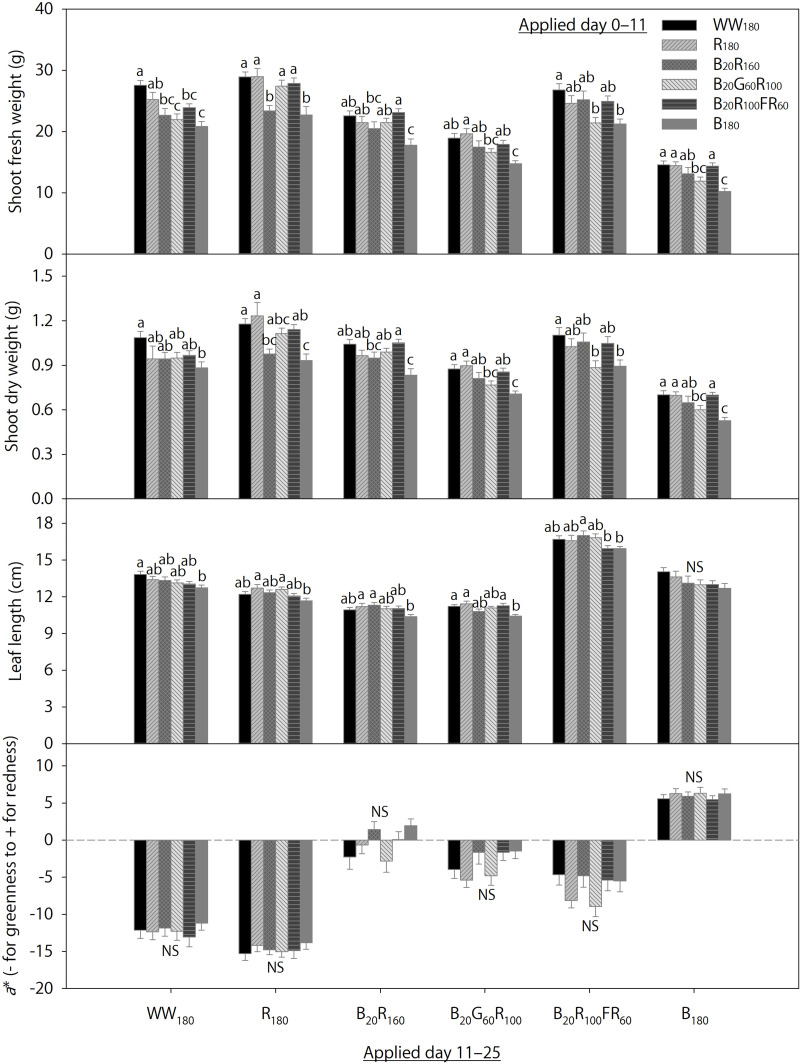
Shoot fresh and dry weights, leaf length, and the a* color space coordinate of lettuce “Rouxai” on day 25. Plants were grown under each of six lighting treatments delivered by warm-white (WW) or mixed blue (B; 400–500 nm), green (G; 500–600 nm), red (R; 600–700 nm), and far-red (FR; 700–800 nm) light-emitting diodes (LEDs) during day 0–11, transferred to all six treatments on day 11, and grown until day 25. The number for each LED type is its photon flux density in μmol⋅m^–2^⋅s^–1^. Means followed by different letters within each parameter and treatment applied during day 11–25 are significantly different based on Tukey’s honestly significant difference test (α = 0.05). NS, non-significant. Error bars show standard errors.

Data on day 25 from 36 temporal lighting combinations are shown in Figure 4. Within each eventual treatment applied day 11–25, the initial treatments applied day 0–11 significantly influenced final shoot fresh and dry weights and leaf length on day 25, but not foliage red-green coloration. Irrespective of the eventual treatment, final shoot fresh and dry weights were generally the greatest when plants were initially grown under WW_180_, R_180_, or B_20_R_100_FR_60_ and the lowest when initially grown under B_180_. Responses of final shoot fresh and dry weights to initial treatments B_20_R_160_ and B_20_G_60_R_100_ were variable within each eventual treatment. Final leaf length within each eventual treatment was mostly similar under initial treatments except B_180_, under which final leaf length within each eventual treatment was slightly lower than that under some other initial treatments.

### The Effects of Initial and Eventual Lighting Treatments on Mature Lettuce

**FIGURE 5:**
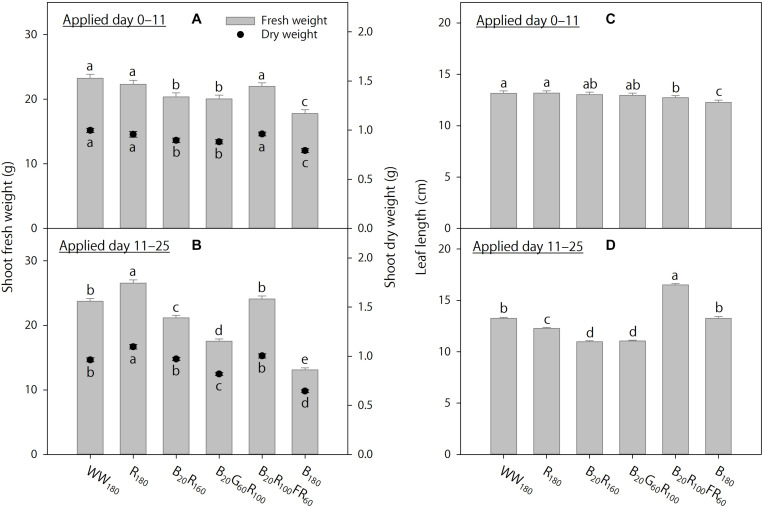
The effects of initial (applied day 0–11) and eventual (applied day 11–25) lighting treatments on pooled final shoot fresh and dry weights and leaf length of lettuce “Rouxai” on day 25. Plants were grown under each of six lighting treatments delivered by warm-white (WW) or mixed blue (B; 400–500 nm), green (G; 500–600 nm), red (R; 600–700 nm), and far-red (FR; 700–800 nm) light-emitting diodes (LEDs) during day 0–11, transferred to all six treatments on day 11, and grown until day 25. The number for LED type is its photon flux density in μmol⋅m^–2^⋅s^–1^. Means followed by different letters within each parameter and graph are significantly different based on Tukey’s honestly significant difference test (α = 0.05). Error bars show standard errors.

**FIGURE 6:**
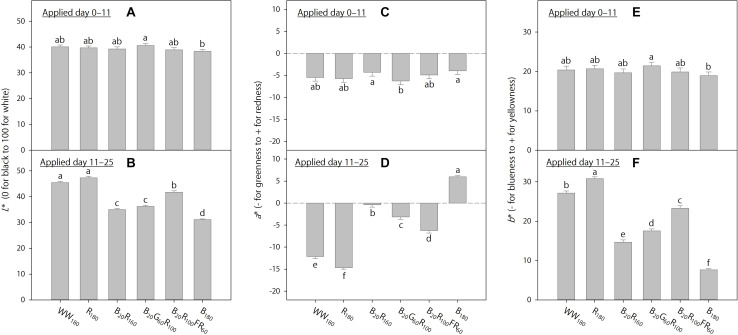
The effects of initial (applied day 0–11) and eventual (applied day 11–25) lighting treatments on pooled final Lab color space parameters of lettuce “Rouxai” on day 25. Plants were grown under each of six lighting treatments delivered by warm-white (WW) or mixed blue (B; 400–500 nm), green (G; 500–600 nm), red (R; 600–700 nm), and far-red (FR; 700–800 nm) light-emitting diodes (LEDs) during day 0–11, transferred to all six treatments on day 11, and grown until day 25. The number for each LED type is its photon flux density in μmol⋅m^–2^⋅s^–1^. Means followed by different letters within each graph are significantly different based on Tukey’s honestly significant difference test (α = 0.05). Error bars show standard errors.

To dissect the effects of initial (applied day 0–11) and eventual (applied day 11–25) lighting treatments on lettuce harvested on day 25, data of plants grown under the same initial treatments were pooled for initial treatment analysis, whereas data of plants grown under the same eventual treatments were pooled for eventual treatment analysis. The effects of the six lighting treatments on final shoot fresh and weight weights, leaf length, and color parameters were different when applied day 0–11 versus day 11–25 (Figures 5,6).

When the lighting treatments were applied day 0–11, final shoot fresh and dry weights (on day 25) were the greatest under WW_180_, R_180_, and B_20_R_100_FR_60_, followed by B_20_R_160_ and B_20_G_60_R_100_, and the lowest under B_180_ (Figure 5A). In addition, final leaf length under WW_180_ and R_180_ was slightly greater than that under B_20_R_100_FR_60_ and B_180_ (Figure 5C). Leaves were slightly brighter under B_20_G_60_R_100_ than under B_180_, slightly redder under B_20_R_160_ and B_180_ than under B_20_G_60_R_100_, and slightly yellower under B_20_G_60_R_100_ than under B_180_ (Figures 6A,C,E). Otherwise, leaf color parameters were similar under most treatments.

Treatment effects were more pronounced when applied day 11–25. Final shoot fresh and dry weights were the greatest under R_180_, followed by WW_180_ and B_20_R_100_FR_60_, and the lowest under B_180_ (Figure 5B). Partially substituting B radiation for R_180_ decreased shoot weight. Substituting 60 μmol⋅m^–2^⋅s^–1^ of G and FR radiation for R radiation in B_20_R_160_ decreased and increased shoot weight, respectively. Final leaf length was the greatest under B_20_R_100_FR_60_, followed by WW_180_ and B_180_, and lowest under B_20_R_160_ and B_20_G_60_R_100_ (Figure 5D). Leaf length under R_180_ was between that under WW_180_ and B_20_R_160_. Leaf color was the brightest under WW_180_ and R_180_, followed by B_20_R_100_FR_60_, and the least bright under B_180_ (Figure 6B). Leaf brightness under B_20_R_160_ and B_20_G_60_R_100_ was between that under B_20_R_100_FR_60_ and that under B_180_. Leaves were the reddest under B_180_, followed by B_20_R_160_, and the least red under R_180_, followed by WW_180_ (Figure 6D). Compared to B_20_R_160_, leaf redness was reduced with substitutional G and FR radiation, especially with the latter. The *b*^∗^ trend was the opposite of the *a*^∗^ trend (Figure 6F).

## Discussion

When lettuce “Rouxai” received static lighting throughout this study, phenotypic responses during the propagation and production phases were generally similar but varied under some treatments. On days 11 and 25, increasing B:R decreased shoot fresh and dry weights, increased leaf redness, and decreased leaf brightness and yellowness. In addition, increasing B:R decreased leaf length on day 11. These results are consistent with the notion that B radiation generally inhibits extension growth and shoot weight while promoting accumulation of chlorophylls, anthocyanins, and other secondary metabolites (Son and Oh, 2013; Kopsell et al., 2015; Wollaeger and Runkle, 2015). However, compared to R_180_, leaf length on day 25 was lower under B_20_R_160_ but similar under B_180_. Aberrant promotion of extension growth and weight gain by B radiation alone was previously observed in cucumber (*Cucumis sativus* L.) and cherry tomato (*Solanum lycopersicum* L. var. *cerasiforme*) seedlings and lettuce “Grand Rapids” (Eskins et al., 1995; Liu et al., 2009; Hernández and Kubota, 2016). We showed a novel temporal shift of the B radiation function from growth inhibition during the seedling phase to promotion of leaf expansion, but not shoot weight, during the production phase of lettuce. Therefore, temporal specificity should be considered at least in some crops when evaluating spectral influence on plant growth.

Extension growth in arabidopsis seedlings is regulated by the activities of cryptochromes 1 and 2, which depend on the B photon flux density (Pedmale et al., 2016). Cryptochromes 1 and 2 interacted with phytochrome-interacting factors 4 and 5 in low B radiation to promote hypocotyl growth, whereas active repression of phytochrome-interacting factor 4 and degradation of cryptochrome 2 and phytochrome-interacting factor 5 in high B radiation restricted it (Pedmale et al., 2016). In the present study, all leaves of lettuce seedlings grown under high B radiation exhibited typical inhibition of extension growth. However, as lettuce matured, layers of newer leaves emerged from the central meristem and covered older ones. The newer leaves were directly exposed to abundant B radiation, whereas the older ones became shaded and received less B radiation (Franklin, 2016). Therefore, the responses and interactions of cryptochromes and phytochrome-interacting factors likely differed in upper and lower leaves, which mostly received high and low B radiation, respectively. The decrease in the incident B photon flux density with leaf maturity could explain the shift from inhibited extension growth of seedlings to promoted extension growth of mature plants under externally static and strong B radiation.

We also observed dynamic growth responses of lettuce to substitutional G radiation. Substituting 60 μmol⋅m^–2^⋅s^–1^ of G radiation for R radiation influenced lettuce shoot weight and leaf length differently on days 11 and 25. It did not affect shoot fresh and dry weights but increased leaf length on day 11. In contrast, it decreased shoot fresh and dry weights but did not affect leaf length on day 25. However, when growing lettuce “Rouxai” under WW radiation for 4 days before varying spectral treatments, plants under B_20_G_60_R_100_ had higher shoot fresh weight, but similar shoot dry weight and leaf length, compared to those under B_20_R_160_ on day 30 or 33 (Meng et al., 2020). In a similar study, substituting 36 μmol⋅m^–2^⋅s^–1^ of G radiation for R radiation in static B_24_R_126_ increased shoot fresh and dry weights and leaf area of lettuce “Waldmann’s Green” on day 28, whereas G radiation alone from fluorescent lamps decreased them (Kim et al., 2004). These discrepancies can at least partly be attributed to adaptive responses to G radiation in photosynthetic acclimation and plant architecture, which could change throughout growth phases based on spectral history, sampling time, and other environmental factors.

We consider two phenomena to explain the shifting responses to G radiation in this study. First, the commonly cited McCree curve shows the spectral region near G radiation had the lowest quantum yield when data were expressed on an absorbed photon basis considering the leaf absorption spectrum (McCree, 1972). However, when the same data were expressed on an incident photon basis without considering the leaf absorption spectrum, the photosynthetic efficacy of incident G radiation was comparable to that of incident B radiation and about half that of incident R radiation (McCree, 1972). Therefore, partial substitution of incident R radiation with incident G radiation could reduce overall photosynthetic efficacy and thus weight gain in some species and cultivars. Indeed, at the same B photon flux density of 15, 30, or 45 μmol⋅m^–2^⋅s^–1^, substituting 15 μmol⋅m^–2^⋅s^–1^ of G radiation for R radiation at a constant PPFD of 150 μmol⋅m^–2^⋅s^–1^ reduced the leaf net photosynthetic rate of lettuce “Green Skirt” without affecting leaf morphology (Kang et al., 2016). In addition, the leaf net photosynthetic rate of lettuce was lower under G radiation alone than under R or B radiation alone, B + R radiation, or B + G + R radiation (Kim et al., 2004; Kang et al., 2016).

Second, when delivered at a sufficiently high photon flux density, G radiation can reverse B-induced growth inhibition and elicit the shade-avoidance response, such as accelerated hypocotyl and petiole elongation (Folta and Maruhnich, 2007; Zhang et al., 2011; Wang and Folta, 2013). In arabidopsis, G radiation reversed activation of cryptochrome 1 and degradation of cryptochrome 2 by B radiation (Bouly et al., 2007). Accumulation of cryptochrome 2 in substitutional G radiation can promote activity of phytochrome-interacting factors 4 and 5 and thus increase extension growth (Pedmale et al., 2016). Besides stem growth, partially substituting G radiation for R radiation in constant B radiation promoted leaf expansion of lettuce “Waldmann’s Green” (Kim et al., 2004), which likely increased light capture for photosynthesis. In addition, completely substituting G radiation for R radiation in B_80_R_80_ increased leaf area of tomato (*S. lycopersicum* L.) seedlings but did not influence shoot fresh or dry weight (Wollaeger and Runkle, 2014), which resembles the lettuce seedling response to G radiation on day 11 in the present study. In other studies, the inclusion of G radiation generally did not influence plant growth (Hernández and Kubota, 2016; Snowden et al., 2016), indicating G radiation effects could depend on the genotype, spectral context, and timing of treatments.

Taken together, the varying responses to substitutional G radiation observed on days 11 and 25 in the present study could be attributed to a changing balance between its reduction of instantaneous photosynthesis and its enhancement of whole-plant photosynthesis through increased leaf expansion and light interception. As lettuce grown under B + R radiation received less overall B radiation later in production because of leaf layering, its sensitivity to additional shade signals such as G radiation (when added) subsided. This could explain why leaf length under B_20_G_60_R_100_ was initially greater than that under B_20_R_160_ on day 11 but eventually was similar to it on day 25. Increased leaf expansion likely compensated for reduced net photosynthesis in G radiation on day 11, leading to comparable shoot weight under B_20_R_160_ and B_20_G_60_R_100_. The lack of such compensation on day 25 resulted in lower shoot weight under substitutional G radiation.

**FIGURE 7:**
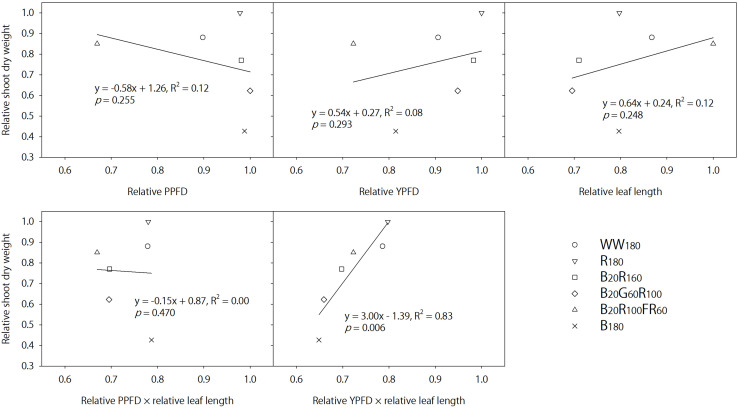
Relative shoot dry weight of lettuce “Rouxai” on day 25 plotted against the relative photosynthetic photon flux density (PPFD), relative yield photon flux density (YPFD), relative leaf length, relative PPFD × relative leaf length, and relative YPFD × relative leaf length. Plants were grown under six static lighting treatments delivered by warm-white (WW) or mixed blue (B; 400–500 nm), green (G; 500–600 nm), red (R; 600–700 nm), and far-red (FR; 700–800 nm) light-emitting diodes (LEDs). The number for each LED type is its photon flux density in μmol⋅m^–2^⋅s^–1^. Data were averaged for each lighting treatment from two blocks. Linear regression equations, coefficients of determination, and p-values for slopes are provided. The only significant linear relationship occurs between relative YPFD × relative leaf length and relative shoot dry weight (α = 0.05).

In contrast, FR radiation was a stronger shade signal than G radiation at the same photon flux density (Meng et al., 2019) and consistently increased leaf length by 41–42% on days 11 and 25 when added to B + R radiation. Lettuce grown under B_20_R_100_FR_60_ had similar shoot dry weight and 17–22% higher shoot fresh weight (partly due to increases in moisture content) compared to that under B_20_R_160_, although B_20_R_100_FR_60_ was 32% lower in the PPFD and 26% lower in the YPFD. The similar TPFDs across all lighting treatments cannot explain differences in shoot dry weight. In addition, Figure 7 plots shoot dry weight against the relative PPFD, YPFD, leaf length, PPFD × leaf length, or YPFD × leaf length for all lighting treatments. Only YPFD × leaf length was linearly related with shoot dry weight (Figure 7). Therefore, the similar dry weight with the FR radiation substitution (B_20_R_100_FR_60_ versus B_20_R_160_) was likely the product of the reduced YPFD (74% of that for B_20_R_160_) and increased light interception (141–142% of that under B_20_R_160_). This suggests that changes in shoot dry weight can be predicted by multiplying percentage changes in the YPFD (to account for the changing instantaneous photosynthetic rate and quantum efficiency) and percentage changes in leaf size (to account for changing light interception due to morphological acclimation). The YPFD is a better predictor of plant biomass than the PPFD because it accounts for relative quantum efficiency and the contribution of FR radiation to net photosynthesis, albeit less significant than B, G, or R radiation. Lastly, light interception may be better estimated with leaf area instead of leaf length.

Increasing B:R intensified red coloration of lettuce “Rouxai,” whereas substitutional G or FR radiation decreased B-induced anthocyanin accumulation of plants treated with static lighting on days 11 and 25. Similarly, increasing the B photon flux density from 20 to 80 μmol⋅m^–2^⋅s^–1^ increased anthocyanin concentration of lettuce “Red Sails” in a dose-dependent manner; however, the inclusion of G radiation reduced anthocyanin accumulation in lettuce “Red Sails” and arabidopsis (Zhang and Folta, 2012). Upregulation of anthocyanin accumulation by B radiation is mediated by cryptochrome 1 and reversed by G radiation (Bouly et al., 2007). Although FR radiation increases anthocyanin accumulation during de-etiolation of arabidopsis seedlings through phytochrome A, which stabilizes Long Hypocotyl 5 (HY5) to promote expression of anthocyanin biosynthetic genes (Li et al., 2014; Liu et al., 2015), it can also decrease anthocyanin accumulation through phytochrome B (Zheng et al., 2013). In addition, partial substitution of white radiation with FR radiation decreased anthocyanin concentration of lettuce “Red Cross” (Li and Kubota, 2009). Therefore, G and FR radiation likely antagonize B radiation in regulation of anthocyanin accumulation of red-leaf lettuce through cryptochromes and phytochromes, respectively. Alternatively, with similar total anthocyanin content per leaf, anthocyanin concentration can decrease as leaf area increases with G or FR radiation. Direct biosynthetic regulation and the “dilution” effect could occur concurrently and warrant further investigation.

When lettuce “Rouxai” was grown under different initial treatments day 0–11 but the same eventual treatments day 11–25, initial light quality had a residual effect on final shoot fresh and dry weights, responses of which generally resembled those under static treatments. For example, for plants transferred to the same R_180_ or B_180_ treatment on day 11, final shoot dry weight was greater when initially grown under R_180_ than under B_180_. In a similar study, when lettuce “Grand Rapids” was transferred from R_100_ to B_100_ or from B_100_ to R_100_ on day 7, shoot dry weight of mature lettuce was primarily influenced by light quality applied before, rather than after, the transfer (Eskins et al., 1995). Contrary to typical B-induced growth inhibition, shoot dry weight and leaf area were consistently greater under B_100_ than under R_100_ applied during seedling development or throughout the experiment (Eskins et al., 1995). Such unique B radiation responses may be species- and cultivar-specific. In addition, a temporal shift of B radiation responses was previously reported in lettuce “Banchu Red Fire,” which was grown under fluorescent lamps day 0–10; R_100_, B_50_R_50_, or B_100_ day 10–17; and then sunlight with supplemental fluorescent lamps day 17–45 (Johkan et al., 2010). Increasing B:R during the seedling phase decreased leaf area and fresh weight on day 17 but increased them on day 45 (Johkan et al., 2010). Although spectral effects varied in these and our studies, they all showed lasting influences of light quality applied during the seedling phase on subsequent plant growth. A sustained environmental treatment delivered early in seedling development could persist into the mature phase possibly by DNA methylation or irreversible activation or suppression of growth-related genes (Bird, 1993; Eskins et al., 1995). The latency of early light signals was also evident in accelerated flowering of mature snapdragon and petunia (*Petunia* × *hybrida* L.) by additional FR radiation applied during the seedling phase (Park and Runkle, 2017, 2018).

Although light quality during the seedling phase modified shoot fresh and dry weights of mature lettuce, the magnitude of this modification was less pronounced than that by light quality in the mature phase. As the plant underwent the exponential growing phase, light interception increased drastically with leaf development, which likely led to greater impacts of eventual treatments on photosynthesis and morphology. In addition, leaf length and coloration of mature lettuce were primarily controlled by eventual lighting treatments and negligibly affected by initial ones. The greater influence of eventual light quality on foliage coloration could at least partly be attributed to rapid anthocyanin accumulation in lettuce under light stresses within days (Owen and Lopez, 2015). In general, final lettuce shoot weight, leaf length, and coloration were similar under lighting treatments applied day 0–25 and day 11–25, further highlighting the predominant role of eventual light quality. Nonetheless, the lasting initial spectral effects exerted significant influence on final shoot weight and thus should be considered for growth of both seedlings and mature plants. In another treatment-switching experiment, spectral effects during the seedling (day 0–14) and mature (day 14–28) phases on final growth of lettuce “Crispa” depended on specific lighting combinations (Chang and Chang, 2014). Therefore, dynamic lighting strategies should be based on specific cultivars and potentially interactive environmental factors such as light quality, the PPFD, and temperature.

The following conclusions are in response to the three original hypotheses. First, effects of light quality applied during the seedling phase persisted into the mature phase, although they were less pronounced than those applied during the mature phase. Second, substituting substantial G radiation for R radiation did not influence growth of seedlings but decreased growth of mature lettuce. Third, B radiation alone decreased lettuce shoot weight during both the seedling and mature phases. However, compared to R radiation, B radiation alone suppressed leaf elongation during the seedling phase but promoted it during the mature phase. In addition to testing hypotheses, we conclude temporally alternating light quality improved precision of phenotype control over static lighting. Thus, differential lighting treatments could be delivered at various developmental stages to optimize crop growth and quality attributes. Our results suggest that lettuce biomass can be maximized with WW, R, or B + R + FR radiation during propagation, followed by R radiation during production. End-of-production B radiation can be used to induce anthocyanin accumulation.

## Data Availability Statement

The raw data supporting the conclusions of this article will be made available by the authors, without undue reservation.

## Author Contributions

QM conceived and performed the experiment, collected and analyzed data, and wrote the first draft of the manuscript. ER was the recipient of funds and reviewed experimental design. Both authors contributed to the manuscript revision and approved the final version.

## Conflict of Interest

The authors declare that the research was conducted in the absence of any commercial or financial relationships that could be construed as a potential conflict of interest.
